# Plasma Free Fatty Acids and Metabolic Effect in Type 2 Diabetes, an Ancillary Study from a Randomized Clinical Trial

**DOI:** 10.3390/nu13041145

**Published:** 2021-03-31

**Authors:** Joanna Mitri, Shaheen Tomah, Jeremy Furtado, Mhd Wael Tasabehji, Osama Hamdy

**Affiliations:** 1 Joslin Diabetes Center, 1 Joslin Place, Boston, MA 02215, USA; Shaheen.Tomah@joslin.harvard.edu (S.T.); MhdWael.Tasabehji@joslin.harvard.edu (M.W.T.); Osama.Hamdy@joslin.harvard.edu (O.H.); 2 Department of Nutrition, Harvard T. H. Chan School of Public Health, 665 Huntington Avenue, Boston, MA 02115, USA; jfurtado@hsph.harvard.edu

**Keywords:** fatty acids, dairy, diabetes

## Abstract

Most nutrition studies looking at the association of food with cardiometabolic markers rely on food frequency questionnaires, which are prone to recall bias. Pentadecanoic acid, heptadecanoic acid and trans-palmitoleic acid are fatty acids that are not synthesized endogenously but are obtained from the diet, particularly dairy, making them reasonable biomarkers of dairy consumption. We investigated the association of dairy fatty acid biomarkers with glycated hemoglobin (HbA1c) and cardiovascular risk factors in type 2 diabetes (T2D). In a clinical trial, 111 participants with T2D (age 58.5 ± 8.9 years, HbA1c 8.09 ± 0.96%) were randomized into three groups: a control group that maintained baseline dairy intake, a low-fat (LF) group that incorporated ≥3 servings/day of LF dairy and a high-fat (HF) group that incorporated ≥3 servings/day of HF dairy. We compared the fatty acids (FA) composition between the three groups at 24 weeks. Pentadecanoic acid and trans-palmitoleic acid increased in the HF group by 14.1% ± 5.4% and 17.5% ± 5.1%, respectively, but not in the control and LF groups (*p* = 0.0474 and *p* = 0.0025 for group-by-time interaction, respectively). Those increases were positively associated with changes in total cholesterol, very-low-density lipoprotein cholesterol VLDL-C and triglycerides but were not associated with changes in HbA1c from baseline to 24 weeks. These results suggest that the intervention was successful and that participants were likely compliant, which supports the validity of the main trial.

## 1. Introduction

Previous reports suggest that dietary fat and saturated fat, in particular, are contributing factors to cardiovascular disease (CVD) and can cause insulin resistance. However, it is suggested that not all saturated fats have similar cardiometabolic effects. Most observational studies have shown that full-fat dairy is not associated with higher risk of diabetes. [1,2] In a meta-analysis of randomized controlled clinical trials with healthy subjects, increased dairy food consumption for more than one month led to an increase in weight but had no effect on glucose, lipid profile or blood pressure [3]. Most nutrition studies rely on dietary recall and measure dairy intake with food frequency questionnaires. However, with the self-reporting of dietary information, there are errors and risk of reporting bias. Moreover, dairy used in mixed food may not be accurately captured. Therefore, relying on biomarkers of dairy intake may lead to a better estimate of dairy intake.

Odd-chain fatty acids; 15:0 (pentadecanoic acid) and 17:0 (heptadecanoic acid), and natural trans-fatty acid trans-palmitoleate (C16:1 trans-n-7) are not endogenously synthesized and are obtained from diet, particularly from dairy products, making them potentially useful biomarkers of dairy fat consumption. In addition to their roles as biomarkers, it is possible that these fatty acids may have metabolic effects. Similar to saturated fat, observational studies suggest that not all saturated fatty acids have similar metabolic effects. For example, odd-chain saturated fatty acids (pentadecanoic acid and heptadecanoic acid]) were found to be inversely associated with incident type 2 diabetes in several studies [4,5,6,7,8]. Trans-palmitoleate has been associated with lower insulin resistance, presence of atherogenic dyslipidemia and incident diabetes [7].

In a three-arm clinical trial in patients with type 2 diabetes, we randomly assigned the participants into three diet groups: one group was asked to keep baseline dairy intake and served as the control group; a low-fat (LF) group that included ≥3 servings/d of LF dairy, and a high-fat (HF) group that included ≥3 servings/d of HF dairy. At the end of 24 weeks, there was no significant effect on glycated hemoglobin (HbA1C), lipid profile, blood pressure or body mass index when comparing either the LF or HF group to the control group. In that trial, we relied on dietary recalls to confirm patient compliance.

We hypothesized that biomarkers of dairy intake, specifically pentadecanoic acid (15:0), heptadecanoic acid (17:0) and trans-palmitoleate (C16:1 trans-n-7), will not be associated with higher HbA1c or other cardiometabolic risk factors at the end of the trial.

In this ancillary study, we aimed to look at the association between individual fatty acids, specifically pentadecanoic acid (15:0), heptadecanoic acid (17:0) and trans-palmitoleic acid (trans-16:1n-7), and HbA1c, body weight and cardiovascular risk factors in people with type 2 diabetes. We also investigated the compliance within the controlled clinical trial, looking at specific dairy fatty acid biomarkers: pentadecanoic acid (15:0), heptadecanoic acid (17:0) and trans-palmitoleic acid (trans-16:1n7) at the start and at the end of the trial in each of the three diet groups.

## 2. Materials and Methods

### 2.1. Study Design and Population

This is a secondary analysis of a randomized clinical trial that examined changes in plasma fatty acids and their association with glycemic control and other CVD risk factors in adults with type 2 diabetes. The primary trial was designed to investigate the effect of low-fat and high-fat dairy consumption on glycemic control and other CVD risk factors in patients with type 2 diabetes. The study protocol was approved by the Joslin institution’s committee on human studies (2016-04), and informed consent forms were signed prior to any trial-related activity. This trial was registered at clinicaltrials.gov as NCT02895867.

The study design, methods and results have been published previously [9]. In brief, the study included 111 randomized subjects. The main inclusion criteria included a diagnosis with type 2 diabetes for ≥3 months; glycated hemoglobin (HbA1c) >7%; BMI ≥25 kg/m^2^; consumption of <3 servings/d of dairy products. Outcomes were assessed at three time points: baseline, 12 weeks and 24 weeks. The primary outcome of the trial was the change in HbA1c between the three different dietary intervention groups with varying amounts of dairy intake and dairy fat content. Clinical and laboratory tests were conducted at baseline, 12 weeks and 24 weeks.

### 2.2. Biospecimen Collection

Details on the assays used to collect venous whole-blood samples were reported previously [9]. Briefly, fasting samples were collected using a BD Vacutainer^®^ serum separator tube (SST™, BD) (BD, Franklin Lakes, NJ, USA) and then centrifuged at 1100–1300× *g* for 10 min to separate serum. Then, 1 mL of serum was aliquoted in a separate cryogenic vial and immediately stored in at −80 °C for later analysis of fatty acids. Samples were collected at baseline, 12 weeks and 24 weeks.

### 2.3. Free Fatty Acids Analysis

Fatty acids were determined as previously described by Baylin et al. [8]. Briefly, fatty acids were extracted and trans-methylated with methanol and sulfuric acid as described by Zock et al. [10,11]. After esterification, the fatty acid methyl esters were re-dissolved in iso-octane and quantitated using gas–liquid chromatography. Hydrogen was used as a carrier gas (1.3 mL/min flow rate) and the injection port was heated to 240 °C and was set to splitless mode. The GC column was a fused silica capillary cis/trans column (SP2560; Supelco, Bellefonte, PA, USA) that was 100 m in length, 0.25 μm in internal diameter and had a 0.20-μm stationary phase. The gas chromatograph was a Hewlett-Packard GC 6890 (Palo Alto, CA, USA) equipped with a flame ionization detector (FID) Samples were injected using a Hewlett-Packard 7673 Autosampler injector. The sample injection volume was 1 μL. The GC oven started at a temperature of 90 °C and then rose to 170 °C at a rate of 10 °C/min. The temperature was maintained at 170 °C for 5 min. Then, the temperature rose at a rate of 5 °C/min from 170 to 175 °C, followed by a rate of 2 °C/min from 175 to 185°C, a rate of 1 °C/min from 185 to 190 °C and a rate of 5 °C/min from 190 to 210 °C. The oven temperature was held constant at 210 °C for 5 min. Then, the temperature increased at a rate of 5 °C/min from 210 to 250 °C and was held constant at 250 °C for 10 min. Known standards (purity >99%; NuCheck Prep, Elysium, MN, USA) were used to establish peak retention times. Instruments were operated and chromatography was integrated using Agilent Technologies ChemStation A.08.03. Two duplicate control samples were run in each batch to monitor quality control. The lab participates in external validation programs offered by the National Institute of Standards and Technology and the American Oil Chemists Society.

### 2.4. Statistical Analyses

Study data were collected and managed using Research Electronic Data Capture (REDCap). The intent-to-treat (ITT) principle was used to analyze study endpoints by including all randomly assigned subjects. Multiple imputations were used to handle missing values in the ITT analysis (SAS PROC MI procedure). To analyze the study endpoints, we constructed a linear mixed-effects model (ANOVA; PROC MIXED) with group, time and group-by-time interaction as fixed effects and subject as the random effect. Adjusting for baseline HbA1c, homeostatic model assessment of insulin resistance (HOMA-IR), age or sex did not affect the outcomes. Therefore, results are reported for the unadjusted model. We used linear contrasts within the SAS Mixed Procedure (PROC MIXED) for cross-sectional comparisons between study groups. Univariate and multivariate linear regression models were used to explore the association between changes in plasma levels of free fatty acids (FFA) and study outcomes while adjusting for candidate covariates. A 2-sided *p* < 0.05 was considered statistically significant. Statistical analyses were performed using SAS version 9.4 (SAS Institute, Inc., Cary, NC, USA) (mixed-effects model) or STATA SE 15.0 (StataCorp) (linear regression).

## 3. Results

### 3.1. Main Study Results

In total, 111 participants with type 2 diabetes were enrolled (aged 58.5 ± 8.9 y, 47% females, diabetes duration 13.2 ± 8.3 y, HbA1c 8.09 ± 0.96%). The results have been published elsewhere [9]. Briefly, at 24 weeks, the percent energy from saturated fat increased from baseline in the HF group by 3.6% (95% CI: 2.2, 5.1) and decreased in the LF group by −1.9% (95% CI: −3.3, −0.4). The LF group increased their percent energy from protein by 4.5% (95% CI: 2.6, 6.4). The HF group decreased their percent energy from carbohydrates by −3.4% (95% CI: −0.2, −6.7). There were no differences in the mean changes in HbA1c, body weight, BMI, body composition or lipid parameters or BP between the three groups at 24 weeks.

### 3.2. Plasma Fatty Acids Results

#### 3.2.1. Changes in Plasma Fatty Acid Profile after Dietary Interventions

**Figure 1:**
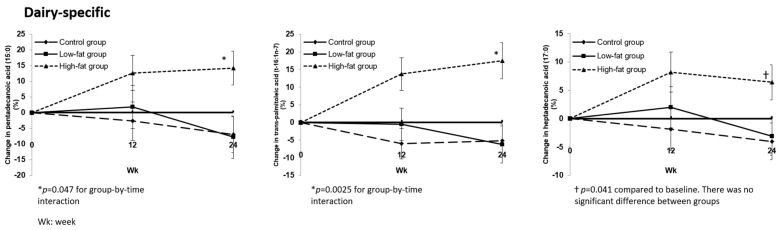
Percent change in dairy specific plasma fatty acids among study groups. Data are least-square mean difference ± SEM. Intent-to-treat population: control group *(n* = 38); low-fat group (*n* = 36); high-fat group (*n* = 37).

At the end of 24 weeks, six plasma fatty acids were different among the three groups. Of the known dairy biomarkers, both pentadecanoic acid (15:0) and trans-palmitoleic acid (t-16:1n-7) increased from baseline in the HF group by 14.1% ± 5.4% (*p* = 0.011) and 17.5% ± 5.1% (*p* = 0.0019), respectively, compared to the control and LF groups (*p* = 0.0474 and *p* = 0.0025 for group-by-time interaction, respectively) (Figure 1).

**Figure 2:**
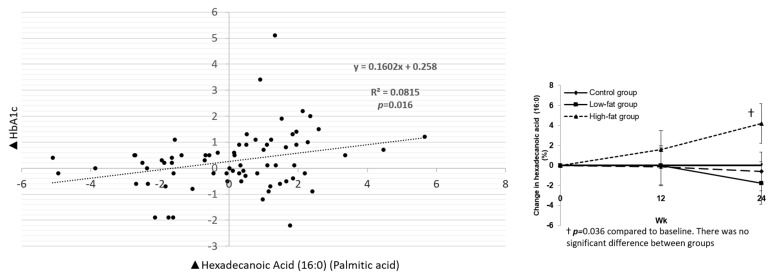
Association between change in plasma levels of hexadecanoic acid (16:0) (palmitic acid) and change in glycated hemoglobin (HbA1c).

Both heptadecanoic acid (17:0) and hexadecanoic acid (palmitic acid) (16:0) increased in the HF group by 6.4% ± 3.1% and 4.2% ± 2%, respectively, from baseline (*p* = 0.0413 and *p* = 0.036, respectively), but this change was not significantly different from the other diet groups at 24 weeks, *p* = 0.111 and *p* = 0.25, respectively (Figure 1 and Figure 2).

Among the other fatty acids detected that are not considered biomarkers of dairy intake, the saturated fatty acid nonadecanoic acid (19:0) increased by 20.21% ± 9.31% in the HF group compared to an increase of 1.15% ± 9.51% in the LF group and a decrease of −12.41 ± 8.37% in the control group (*p* = 0.015 for group-by-time interaction) (Table S1)

**Figure 3:**
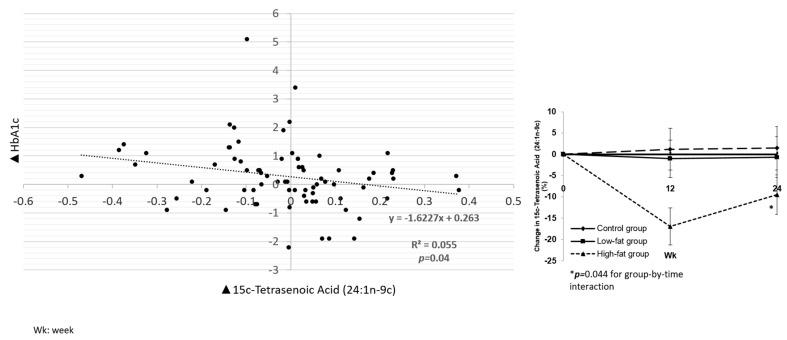
Association between change in plasma levels of 15c-Tetrasenoic Acid (24:1n−9c) and change in HbA1c.

Nervonic acid (15c-tetrasenoic acid, selacholeic acid) (24:1n−9c) was the only monounsaturated fatty acid in which we noted a change: a decrease from baseline in the HF arm by 9.6% ± 4.1% with no change in the LF and control groups (*p* = 0.04) (Figure 3).

The trans-fatty acid 9c,12t-octadecadienoic acid (18:2n-6ct) increased by 12.1% ± 3.6% in the HF group at 24 weeks, while it decreased in the LF and control groups (−1.6% ± 3.8% and −0.7% ± 3.5, respectively; *p* = 0.0009 for group-by-time interaction) (Table S1)

Furthermore, 9c,11t-octadecadienoic acid (18:2n−7c) increased by 20.8% ± 5.8% in the HF group at 24 week, while it decreased in the LF and control groups (−3.27% ± 5.8% and −3.25% ± 5.32%, respectively; *p* = 0.002 for group-by-time interaction) (Table S1).

There was no difference between groups in polyunsaturated fatty acids at the end of the intervention (Table S1).

#### 3.2.2. Associations between Fatty Acids and Cardiometabolic Biomarkers

In the regression analysis, none of the dairy biomarkers (pentadecanoic acid (15:0), heptadecanoic acid (17:0) or trans-palmitoleic acid (t-16:1n-7)) were associated with HbA1c.

Associations between other fatty acids not considered markers of dairy intake with HbA1c, triglycerides (TG),low-density lipoprotein cholesterol (LDL-C) and VLDL-C are shown in Table S2. Of note, hexadecanoic acid (palmitic acid) (16:0) was positively associated with HbA1c (*p* = 0.016) (Figure 2), while 15c-tetrasenoic acid (nervonic acid) (24:1n−9c) was inversely associated with HbA1c (*p* = 0.04) (Figure 3). Both docosanoic (behenic) acid (22:0) and tetracosanoic (lignoceric) acid (24:0) were inversely associated with HbA1c (Table S2).

Pentadecanoic acid (15:0), hexadecanoic acid (palmitic acid) (16:0), heptadecanoic acid (17:0), trans-palmitoleic acid (t-16:1n-7), nonadecanoic acid (19:0), 9c,11t-octadecadienoic acid (18:2n−7c) and 9c,12t-octadecadienoic acid (18:2n−6ct) were positively associated with VLDL-C and triglycerides, whereas only 11t-octadecadienoic acid (18:2n−7c) was positively associated with total cholesterol, and 15c-tetrasenoic acid (24:1n−9c) (nervonic acid) was inversely associated with TG and VLDL-C (Table S2).

None of the changes in fatty acids were associated with weight or blood pressure (data not shown).

## 4. Discussion

In this ancillary study, an increase in high-fat dairy consumption to ≥3 servings/day compared to <3 servings/day while maintaining energy intake was associated with an increase in pentadecanoic acid (15:0) and trans-palmitoleic acid (t-16:1n-7) in patients with type 2 diabetes. Heptadecanoic acid (17:0), another dairy biomarker, was also increased in the high-fat dairy arm, but the change was not statistically significant. Those three dairy fatty acids were not associated with HbA1c, but they were positively associated with TG and VLDL-C. We have previously shown that in patients with type 2 diabetes, an increase in dairy consumption to ≥3 servings/day compared to <3 servings/day in an isocaloric diet, irrespective of its fat content, has no effect on HbA1c, body weight, body composition, lipid profile or BP. This study found that the fatty acid biomarkers of dairy intake increased in the high-fat dairy arm; therefore, the lack of effect found in the abovementioned study was not due to non-compliance.

The fatty acid biomarkers of dairy actually make up a small proportion of the fat in milk; the proportions of fat for 15:0, 17:0 and 16:1-trans are 1.1%, 0.6% and 0.3%, respectively. Despite these low proportions, our intervention was associated with a significant increase in the levels of those fatty acids. This confirms previous studies that suggest that there is utility in these three fatty acids as biomarkers of dairy intake despite their low relative abundance in both plasma and milk.

There is a persistent notion that saturated fatty acids should be avoided generally. This is likely due to the known effect of saturated fat on insulin resistance and incidence of type 2 diabetes [12,13]. Not all saturated fatty acids have the same biological effects. In a Swedish cohort, the highest quintile of regular-fat dairy consumption was associated with a 23% lower risk of type 2 diabetes (T2D) in comparison to the lowest quintile [14]. In addition, it is important to note that we do not consume specific nutrients in isolation but rather consume them as part of complex foods. Therefore, although a specific saturated fatty acid may have a beneficial or harmful effect, complex foods may have different influences. Dairy food is a good example of this. Though high in saturated fat with myristic (14:0), palmitic (16:0) and stearic (18:0) acids comprising over 50% of the total fat content, a review of several studies found no increase in risk of T2D with increased intake of dairy products [15].

Odd-chained saturated fatty acids (SFAs) and very long saturated fatty acids may have protective effects, whereas even-chained SFAs may have detrimental effects [16,17]. In regard to dairy fatty acids, most of the previous studies have shown either a neutral association or a beneficial effect on incidence of diabetes. In multivariate analyses, FFA 15:0 and FFA 17:0 were inversely associated with fasting plasma glucose [18]. In two separate cohorts of US men and women, three plasma biomarkers of dairy fat, namely 15:0, 17:0, and trans-16:1n-7, were associated with a lower risk of incident diabetes mellitus [5]. Dairy fat intake, on the other hand, has been associated with glucose tolerance, hepatic and systemic insulin sensitivity and liver fat but not β-cell function in humans [18].

Circulating and tissue biomarker concentrations of odd-chain saturated fats (15:0, 17:0) and natural ruminant trans-fats (trans-16:1n-7) partly reflect dairy fat consumption, help capture multiple dietary sources without relying on memory or subjective reporting and reflect a complementary approach to look at compliance with dairy food interventions.

In the Insulin Resistance Atherosclerosis Study (IRAS) cohort, saturated fatty acids that are even-chained and shorter in length (14:0 and 16:0) were positively related to pro-inflammatory markers. Longer even-chained SFAs (20:0 and 22:0) and an odd-chained SFA (15:0) had inverse associations [19].

It is not surprising to see the inverse association of both docosanoic (behenic) acid (22:0) and tetracosanoic (lignoceric) acid (24:0) with HbA1c as higher plasma concentrations of very-long-chain saturated fatty acids have been associated with lower risk of type 2 diabetes [20].

Most of the studies that looked at the association of dairy fatty acids with dairy intake relied on dietary recall [5]. However, dairy fat is consumed not just as a whole food only but mixed into numerous foods. Food frequency questionnaires that estimate dairy fat intakes from whole foods and major mixed sources may not accurately capture quantities in mixed food. In our study, we have seen an increase in dairy free fatty acids directly associated with the intervention, providing stronger evidence that these fatty acids could serve as biomarkers of dairy fat.

In the main study results, we did not see a significant difference between the three groups at 24 weeks in the mean changes in HbA1c, body weight, BMI, body composition or lipid parameters or BP; however, there was a trend of higher HbA1c in the LF and HF groups. It is possible that higher HbA1c with dairy could be confounded by increased carbohydrate intake in the LF group only as we saw an increase in the total energy expenditure from carbohydrates in the LF group in the main trial. Although dairy is often sweetened, dairy-based desserts or cream did not count toward the three daily servings of dairy in the dairy groups. In this ancillary analysis, while biomarkers of dairy fat were not associated with HbA1c, palmitic acid (16:0) was positively associated with HbA1c, and 15c-tetrasenoic acid (24:1n−9c) was negatively associated with HbA1c. Palmitic acid is naturally produced at low levels by a wide range of plants and organisms. It is not only present in dairy but can also be found in cocoa butter, soybean oil and sunflower oil. On the other hand, nervonic acid (15c-tetrasenoic acid (24:1n−9c)), a monounsaturated fat present in seed oils of plants, was decreased from baseline in the HF arm by 0.06% in comparison to the low-fat and control groups (*p* = 0.04).

It is interesting that not all saturated fats were associated with higher TG and VLDL-C, only dodecanoic acid (12:0), tetradecanoic acid (14:0), pentadecanoic acid (15:0), hexadecanoic acid (palmitic acid) (16:0), heptadecanoic acid (17:0), nonadecanoic acid (19:0) (Table S2). Based on other studies, this suggests that apoC3 would likely be increased and, together with increases in TG and VLDL-C, would be expected to increase risk of type 2 diabetes and heart disease. Previous analyses have shown a moderate association between fatty acids and levels of triglycerides, and while SFA had a positive association with TG, long-chain polyunsaturated fatty acids (PUFAs) had a negative one [21].

This study is limited by the fact that it is an ancillary study and the association that we found does not imply a true causation. In addition, it has been suggested that trans-palmitoleic acid (trans-16:1, *n*−7) can be synthesized endogenously from vaccenic acid (trans−18:1, *n*−11), which can be present in partially hydrogenated fats and oils [22]. However, one of the major strengths of our study is the use of biomarkers of diary consumption in a clinical control setting, unlike other publications that correlated those biomarkers with self-reported dietary recalls or food frequency questionnaires.

## 5. Conclusions

In conclusion, consumption of ≥3 servings of high-fat dairy per day can be maintained over 24 weeks and has no significant effect on HbA1c. However, the association of biomarkers of fatty acids with higher triglycerides and VLDL-C suggests the need to better understand potential health effects of dairy fat and metabolic determinants of fatty acids especially when dairy fats are replacing other fats in the diet.

## Data Availability

De-identified data presented in this study may be made available on reasonable request from the corresponding author and subject to institutional approval.

## Supplementary Materials

The following are available online at https://www.mdpi.com/article/10.3390/nu13041145/s1, Table S1: Associations between analyzed plasma fatty acids and selected cardiometabolic biomarkers; Table S2: Change in analyzed plasma fatty acids from baseline to 12 and 24 weeks in study groups.
