# Supplementary material for: Plasma Free Fatty Acids and Metabolic Effect in Type 2 Diabetes, an Ancillary Study from a Randomized Clinical Trial
Source: Nutrients. 2021 Mar 31;13(4):1145. doi: 10.3390/nu13041145 (PMC8065525; doi:10.3390/nu13041145)
Supplement: Supplementary file 1 [file nutrients-13-01145-s001.pdf]

Supplement Tables

Supplemental Table 1

Fatty Acid	Control group (n = 38)			Low-fat group (n = 36)			High-fat group (n = 37)			P[1]	P[2]	P[3]
	Baseline	12 wk	24 wk	Baseline	12 wk	24 wk	Baseline	12 wk	24 wk			
**Saturated**												
**Dodecanoic Acid (12:0)**	0.008 (0.005)	0.009 (0.005)	0.017 (0.007)	0.005 (0.005)	0.01 (0.005)	0.005 (0.007)	0.024 (0.005)	0.029 (0.005)	0.03 (0.007)	0.84	0.0018	0.3827
**Tetradecanoic Acid (14:0)**	0.5 (0.05)	0.6 (0.06)	0.6 (0.07)	0.34 (0.05)	0.4 (0.07)	0.33 (0.07)	0.53 (0.05)	0.64 (0.07)	0.62 (0.05)	0.77	0.001	0.056
**Pentadecanoic Acid (15:0)**	0.13 (0.007)	0.13 (0.009)	0.12 (0.008)	0.11 (0.008)	0.12 (0.009)	0.11(0.009)	0.143 (0.007)	0.16 (0.009)	0.163(0.008)	0.047	0.0005	0.35
**Hexadecanoic Acid (16:0)**	20 (0.4)	20 (0.4)	20 (0.5)	19.5 (0.4)	19.5 (0.4)	19.1 (0.5)	19.7 (0.4)	20 (0.4)	20.5 (0.4)	0.25	0.34	0.85
**Heptadecanoic Acid (17:0)**	0.31 (0.009)	0.3 (0.01)	0.29 (0.01)	0.32 (0.009)	0.33 (0.01)	0.31 (0.01)	0.32 (0.009)	0.35 (0.01)	0.35 (0.01)	0.11	0.019	0.28
**Octadecanoic Acid (18:0)**	7.76 (0.140	7.96 (0.15)	7.86 (0.16)	7.67 (0.14)	7.53 (0.16)	7.39 (0.17)	7.77 (0.14)	7.71 (0.15)	7.69 (0.16)	0.37	0.2	0.46
**Nonadecanoic Acid (19:0)**	0.037 (0.002)	0.039 (0.003)	0.032 (0.003)	0.035 (0.002)	0.038 (0.003)	0.036 (0.003)	0.033 (0.002)	0.046 (0.003)	0.04 (0.003)	0.015	0.36	0.004
**Eicosanoic (Arachidic) Acid (20:0)**	0.266 (0.01)	0.267 (0.01)	0.257 (0.01)	0.246 (0.01)	0.24 (0.01)	0.239 (0.01)	0.231 (0.01)	0.234 (0.01)	0.235 (0.01)	0.65	0.08	0.64
**Docosanoic (Behenic) Acid (22:0)**	0.56 (0.028)	0.54 (0.029)	0.54 (0.031)	0.61 (0.029)	0.55 (0.03)	0.59 (0.033)	0.59 (0.029)	0.55 (0.029)	0.58 (0.032)	0.57	0.65	0.015
**Tricosanoic Acid (23:0)**	0.22 (0.01)	0.23 (0.01)	0.22 (0.01)	0.24 (0.01)	0.23 (0.01)	0.24 (0.01)	0.24 (0.01)	0.23 (0.01)	0.24 (0.01)	0.37	0.72	0.22
**Tetracosanoic (Lignoceric) Acid (24:0)**	0.42 (0.02)	0.42 (0.02)	0.42 (0.03)	0.5 (0.02)	0.45 (0.02)	0.49 (0.03)	0.43 (0.02)	0.36 (0.02)	0.41 (0.03)	0.1	0.04	0.001
**Monounsaturated**												
**9c-Tetradecenoic Acid (14:1n-5c)**	0.021 (0.003)	0.022 (0.004)	0.02 (0.004)	0.015 (0.003)	0.018 (0.004)	0.014 (0.004)	0.023 (0.003)	0.028 (0.004)	0.031 (0.004)	0.54	0.01	0.39
**10c-Pentadecenoic Acid (15:1n-5c)**	0.007 (0.0008)	0.006 (0.0009)	0.006 (0.0008)	0.006 (0.0008)	0.004 (0.0009)	0.005 (0.0009)	0.005 (0.0008)	0.006 (0.0009)	0.007 (0.0009)	0.2	0.36	0.55
**9c-Hexadecenoic Acid (16:1n-7c)**	1.48 (0.1)	1.46 (0.11)	1.47 (0.12)	1.33 (0.1)	1.41 (0.11)	1.38 (0.13)	1.46 (0.1)	1.6 (0.11)	1.69 (0.12)	0.43	0.38	0.25
**9c-Octadecenoic Acid (18:1n-9c)**	21.1 (0.5)	21.4 (0.8)	21.8 (0.6)	21.5 (0.5)	23.5 (0.8)	22.3 (0.7)	21.4 (0.5)	22 (0.8)	22.1 (0.6)	0.46	0.43	0.007
**11c-Octadecenoic Acid (18:1n-7c)**	1.74 (0.04)	1.72 (0.05)	1.71 (0.05)	1.79 (0.04)	1.82 (0.05)	1.82 (0.05)	1.73 (0.04)	1.65 (0.05)	1.6 (0.05)	0.29	0.1	0.32
**11c-Eicosenoic Acid (20:1n-9c)**	0.17 (0.007)	0.16 (0.008)	0.16 (0.007)	0.17 (0.007)	0.19 (0.008)	0.18 (0.008)	0.15 (0.007)	0.16 (0.007)	0.15 (0.007)	0.44	0.01	0.28
**15c-Tetrasenoic Acid (24:1n-9c)**	0.57 (0.04)	0.58 (0.04)	0.58 (0.04)	0.64 (0.04)	0.63 (0.04)	0.64 (0.04)	0.63 (0.04)	0.53 (0.04)	0.58 (0.04)	0.044	0.32	0.085
**Polyunsaturated- Omega-3**												
**9c,12c,15c-Octadecatrienoic Acid (18:3n-3c)**	0.69 (0.04)	0.73 (0.05)	0.74 (0.05)	0.61 (0.04)	0.65 (0.05)	0.7 (0.06)	0.63 (0.04)	0.66 (0.05)	0.64 (0.06)	0.92	0.21	0.24

**5c,8c,11c,14c17c-Eicosapentaenoic Acid (20:5n-3c)**	0.77 (0.08)	0.66 (0.08)	0.63 (0.06)	0.8 (0.08)	0.79 (0.08)	0.82 (0.07)	0.73 (0.08)	0.84 (0.08)	0.78 (0.06)	0.28	0.25	0.86
**7c,10c,13c,16c,19c-Docosapentaenoic Acid (22:5n-3c)**	0.49 (0.02)	0.47 (0.02)	0.47 (0.02)	0.57 (0.02)	0.55 (0.02)	0.6 (0.02)	0.53 (0.02)	0.52 (0.02)	0.53 (0.02)	0.28	0.001	0.12
**4c,7c,10c,13c,16c,19c-Docosahexaenoic Acid (22:6n-3c)**	1.69 (0.1)	1.55 (0.1)	1.58 (0.1)	1.84 (0.1)	1.77 (0.1)	1.72 (0.1)	1.84 (0.1)	1.76 (0.1)	1.76 (0.1)	0.96	0.25	0.08
**Polyunsaturated- Omega-6**												
**9c,12c-Octadecadienoic Acid (18:2n-6cc)**	27.9 (0.64)	27.9 (0.74)	28 (0.8)	27.7 (0.66)	26.3 (0.77)	27.6 (0.87)	27.4 (0.65)	26.7 (0.75)	26.4 (0.82)	0.39	0.41	0.14
**6c,9c,12c-Octadecatrienoic Acid (18:3n-6c)**	0.5 (0.03)	0.5 (0.04)	0.5 (0.03)	0.51 (0.03)	0.53 (0.04)	0.54 (0.04)	0.53 (0.03)	0.57 (0.03)	0.6 (0.03)	0.72	0.35	0.09
**11c,14c-Eicosadienoic Acid (20:2n-6c)**	0.22 (0.007)	0.22 (0.008)	0.22 (0.007)	0.24 (0.007)	0.24 (0.008)	0.25 (0.007)	0.21(0.007)	0.21 (0.008)	0.2 (0.007)	0.59	0.006	0.55
**8c,11c,14c-Eicosatrienoic Acid (20:3n-6c)**	1.62 (0.07)	1.62 (0.08)	1.57 (0.06)	1.73 (0.07)	1.62 (0.08)	1.68 (0.07)	1.64 (0.07)	1.64 (0.08)	1.69 (0.07)	0.28	0.69	0.4
**5c,8c,111c,14c-Eicosatetraenoic Acid (20:4n-6c)**	8.4 (0.4)	8.3 (0.42)	8.3 (0.42)	8.9 (0.4)	8.5 (0.43)	8.8 (0.43)	9.07 (0.39)	8.4 (0.42)	8.3 (0.42)	0.16	0.77	0.007
**13c,16c-Docosadienoic Acid (22:2n-6c)**	0.02 (0.001)	0.02 (0.003)	0.02 (0.001)	0.03 (0.001)	0.03 (0.001)	0.03 (0.004)	0.03 (0.001)	0.04 (0.004)	0.03 (0.002)	0.31	0.0002	0.83
**7c,10c,13c,16c-Docosatetraenoic Acid (22:4n-6c)**	0.3 (0.01)	0.3 (0.02)	0.3 (0.02)	0.33 (0.01)	0.33 (0.01)	0.35 (0.02)	0.33 (0.01)	0.31 (0.01)	0.34 (0.02)	0.09	0.15	0.2
**Trans**												
**9t-Hexadecenoic Acid (16:1n-7t)**	0.12 (0.006)	0.12 (0.006)	0.11 (0.007)	0.12 (0.006)	0.12 (0.006)	0.11 (0.007)	0.11 (0.006)	0.13 (0.006)	0.14 (0.007)	0.0025	0.29	0.41
**6t-Octadecenoic Acid (18:1n-12t)**	0.21 (0.01)	0.22 (0.01)	0.2 (0.02)	0.13 (0.01)	0.11 (0.01)	0.13 (0.02)	0.09 (0.01)	0.12 (0.01)	0.1 (0.02)	0.16	0.0001	0.6
**9t-Octadecenoic Acid (18:1n-9t)**	0.24 (0.01)	0.24 (0.02)	0.22 (0.01)	0.2 (0.01)	0.2 (0.02)	0.2 (0.02)	0.15 (0.01)	0.21 (0.02)	0.17 (0.01)	0.1	0.026	0.23
**11t-Octadecenoic Acid (18:1n-7t)**	0.23 (0.01)	0.25 (0.05)	0.22 (0.02)	0.2 (0.01)	0.22 (0.05)	0.18 (0.02)	0.15 (0.01)	0.25 (0.05)	0.19 (0.02)	0.15	0.53	0.12
**9t,12t-Octadecadienoic Acid (18:2n-6t)**	0.02 (0.001)	0.02 (0.002)	0.02 (0.001)	0.01 (0.001)	0.02 (0.002)	0.02 (0.001)	0.02 (0.001)	0.03 (0.002)	0.02 (0.001)	0.27	0.66	0.049
**9c,12t-Octadecadienoic Acid (18:2n-6ct)**	0.19 (0.006)	0.19 (0.007)	0.19 (0.008)	0.19 (0.006)	0.19 (0.007)	0.19 (0.008)	0.19 (0.006)	0.22 (0.007)	0.21 (0.008)	0.0009	0.22	0.004
**9t,12c-Octadecadienoic Acid (18:2n-6tc)**	0.11 (0.003)	0.11 (0.004)	0.11 (0.008)	0.1 (0.004)	0.09 (0.004)	0.11 (0.008)	0.09 (0.004)	0.1 (0.004)	0.09 (0.008)	0.2	0.025	0.12
**Other**												

Supplement Tables

**9c,11t-Octadecadienoic Acid (18:2n-7c)**	0.1 (0.004)	0.09 (0.006)	0.08 (0.006)	0.09 (0.005)	0.09 (0.006)	0.09 (0.006)	0.09 (0.004)	0.1 (0.006)	0.1 (0.006)	0.002	0.31	0.58

Data are mean normalized % area (SEM).

[1] p for group-by-time interaction

[2] P for group effect

[3] p for time effect

P values calculated using a linear mixed-effects model (analogous to repeated-measures ANOVA; PROC MIXED) with group, visit, and group-by-visit interaction as fixed effects and subject as a random effect.

Supplemental Table 2:

Supplement Tables

Fatty Acid	HbA1c		Total cholesterol		HDL-C		LDL-C		VLDL-C		Triglycerides	
	β (95% CI)	P	β (95% CI)	P	β (95% CI)	P	β (95% CI)	P	β (95% CI)	P	β (95% CI)	P
**Saturated**												
**Dodecanoic Acid (12:0)**	-3.6 (-10.7, 3.5)	0.31	51.4 (-152, 255)	0.61	-11.8 (-49, 26)	0.53	-20.6 (-217, 176)	0.83	77 (11, 143)	0.023	409 (42, 776)	0.029
**Tetradecanoic Acid (14:0)**	0.21 (-0.49, 0.92)	0.54	20.6 (1.13, 40)	0.04	-0.76 (-4.5, 3)	0.68	10.7 (-9, 30)	0.28	10 (3.5, 16.5)	0.003	55.7 (20.5, 90)	0.002
**Pentadecanoic Acid (15:0)**	3.13 (-0.35, 9.8)	0.35	164 (-23, 35)	0.084	17 (-18, 52)	0.34	30 (-160, 220)	0.75	91 (28, 156)	0.005	565 (234, 896)	0.001
**Hexadecanoic Acid (16:0)**	0.16 (0.03, 0.28)	0.015	3.72 (-0.001, 7.4)	0.05	0.33 (-0.38, 1.05)	0.35	0.22 (-3.7, 4.12)	0.9	3.1 (1.96, 4.2)	0.0001	15 (8.78, 21.2)	0.0001
**Heptadecanoic Acid (17:0)**	0.82 (-4.3, 5.9)	0.74	83 (-62, 228)	0.25	13 (-14, 39.8)	0.35	-8 (-153, 137)	0.9	56 (7.1, 105)	0.025	355 (92, 617)	0.009
**Octadecanoic Acid (18:0)**	-0.45 (-0.82, -0.08)	0.016	-8.8 (-19.5, 1.9)	0.1	1.8 (-0.2, 3.8)	0.077	-8.5 (-18.9, 1.8)	0.1	-2.7 (-6.3, 0.9)	0.14	-14.3 (-34, 5.8)	0.16
**Nonadecanoic Acid (19:0)**	-7.2 (-23, 8.8)	0.37	241 (-215, 699)	0.29	53 (-32, 138)	0.21	34 (-481, 413)	0.88	200 (51, 349)	0.009	1166 (355, 1977)	0.005
**Eicosanoic (Arachidic) Acid (20:0)**	-5.75 (-11.6, 0.18)	0.057	-165 (-334, 3.3)	0.055	10.3 (-22, 42)	0.52	-100 (-267, 66)	0.23	-86 (-141, -31)	0.003	-380 (-693, -68)	0.018
**Docosanoic (Behenic) Acid (22:0)**	-2.29 (-4.18, -0.41)	0.018	6.3 (-49, 62)	0.82	4.3 (-6.1, 14)	0.41	36 (-17.4, 89)	0.18	-33 (-50, -16)	0.0001	-161 (-258, -64)	0.001
**Tricosanoic Acid (23:0)**	-8.97 (-13.9, -3.96)	0.001	35 (-120, 190)	0.65	10.7 (-18, 39)	0.46	133 (-12.9, 280)	0.073	-105 (-150, -59)	0.0001	-538 (-797, -279)	0.0001
**Tetracosanoic (Lignoceric) Acid (24:0)**	-3.58 (-5.83, -1.33)	0.002	8.7 (-59, 77)	0.8	5.9 (-6.7, 18.6)	0.35	45 (-19.6, 110)	0.16	042 (-63, -21.9)	0.0001	-209 (-326, -91.8)	0.001
**Monounsaturated**												
**9c-Tetradecenoic Acid (14:1n-5c)**	2.7 (-9.7, 15.1)	0.66	57 (-297, 411)	0.74	6 (-60, 72)	0.85	-207 (-563, 147)	0.24	198 (82, 314)	0.001	1114(510 1719)	0.0001
**10c-Pentadecenoic Acid (15:1n-5c)**	-79 (-140 -19.3)	0.01	-728 (-2524, 1067)	0.42	75 (-260, 410)	0.65	122 (-1612,0.2)	0.88	-932 (-1494, -370)	0.002	-4685 (-7846, -1524)	0.004

Supplement Tables

**9c-Hexadecenoic Acid (16:1n-7c)**	0.5 (-0.04, 1.05)	0.071	10 (-5.4, 26)	0.19	2 (-0.7, 5.1)	0.14	-4.7 (-20.6, 11.1)	0.55	11 (6.1, 15.9)	0.0001	59 (33, 85)	0.0001
**9c-Octadecenoic Acid (18:1n-9c)**	0.12 (0.018, 0.22)	0.021	-0.48 (-3.5, 2.5)	0.75	-0.33 (-0.9, 0.2)	0.25	-2.5 (-5.4, 0.3)	0.085	2.7 (1.7, 3.6)	0.0001	11.8 (6.8, 16.8)	0.0001
**11c-Octadecenoic Acid (18:1n-7c)**	0.99 (-0.09, 2.08)	0.074	-28 (-59, 3.2)	0.079	-6.8 (-12.5 - 1.09)	0.02	-17 (-48, 15)	0.29	-2.2 (-13, 9)	0.7	-31 (-90, 28)	0.29
**11c-Eicosenoic Acid (20:1n-9c)**	2 (-6.2, 10.2)	0.63	-394 (-610, - 177)	0.001	-69 (-109, - 28)	0.001	-399 (-604, - 193)	0.0001	84 (6.7, 161)	0.034	345 (-87, 778)	0.11
**15c-Tetrasenoic Acid (24:1n-9c)**	-1.62 (-3.18,- 0.064)	0.04	-25 (-70, 19.6)	0.26	-2 (-10.8, 6.8)	0.65	16 (-28.5, 60)	0.48	-38 (-51.2,- 24.5)	0.0001	-194 (-267, -121)	0.0001
**Polyunsaturated- Omega-3**												
**9c,12c,15c-Octadecatrienoic Acid (18:3n-3c)**	-0.4 (-1.25, 0.45)	0.35	-16.7 (-40, 7.3)	0.17	-3.5 (-7.9, 0.9)	0.12	-20 (-45, 4.1)	0.1	0.08 (-8.7, 8.9)	0.98	25 (-19.9, 70)	0.27
**5c,8c,11c,14c17c-Eicosapentaenoic Acid (20:5n-3c)**	0.12 (-0.4, 0.66)	0.62	3.8 (-11.3, 19)	0.61	0.9 (-1.9, 3.7)	0.52	6.65 (-7.9, 21)	0.36	-3.9 (-8.9, 1.1)	0.12	-17 (-45, 11)	0.23
**7c,10c,13c,16c,19c-Docosapentaenoic Acid (22:5n-3c)**	-0.65 (-3.28, 1.9)	0.61	-13.7 (-88, 61)	0.71	4.4 (-9.5, 18)	0.53	-0.42(-72, 71)	0.99	-16 (-41, 8.8)	0.2	85 (-223, 53)	0.22
**4c,7c,10c,13c,16c,19c-Docosahexaenoic Acid (22:6n-3c)**	0.34 (-0.19, 0.88)	0.2	-21.6 (-36,-6.9)	0.002	-1.35 (-4.2, 1.5)	0.35	-12.6 (-27, 2)	0.09	-6.8 (-11.8, - 1.8)	0.008	-39 (-66.5, -11.8)	0.006
**Polyunsaturated- Omega-6**												
**9c,12c-Octadecadienoic Acid (18:2n-6cc)**	-0.076 (-.15, - 0.001)	0.046	0.6 (-1.6, 2.7)	0.59	-0.002 (-0.4, 0.4)	0.99	2.1 (0.02, 4.2)	0.048	-1.4 (-2, -0.72)	0.0001	-6.4 (-10.2, -2.6)	0.001
**6c,9c,12c-Octadecatrienoic Acid (18:3n-6c)**	0.06(-1.4, 1.5)	0.93	-1.7 (-44, 41)	0.93	12.9 (5.5, 20.2)	0.001	-25 (-66, 15.5)	0.21	6.7 (-7.8 21)	0.35	49 (-29, 128)	0.21
**11c,14c-Eicosadienoic Acid (20:2n-6c)**	-9.2 (-17 -1.3)	0.023	-250 (-475, -24)	0.03	-48 (-90, -6.3)	0.025	-177 (-405, 50)	0.12	4.6 (-76, 86)	0.9	-179 (-611, 253)	0.41
**8c,11c,14c-Eicosatrienoic Acid (20:3n-6c)**	-1.35 (-2.21, - .049)	0.003	16 (-9.4, 42)	0.2	8.9 (4.5, 13.3)	0.0001	13 (-11, 38)	0.29	-3.8(-12.7, 5)	0.38	-31 (-79, 17)	0.2
**5c,8c,111c,14c-Eicosatetraenoic Acid (20:4n-6c)**	-0.06 (-0.28 0.15)	0.57	-8.8 (-14.8, - 2.8)	0.005	-0.6 (-1.8, 0.5)	0.27	-2.9 (-9.3, 3.4)	0.35	-5 (-6.9, -3.2)	0.0001	-28.6 (-38, -19)	0.0001
**13c,16c-Docosadienoic Acid (22:2n-6c)**	16 (-20, 52)	0.37	-741 (-1774, 290)	0.15	63 (-131, 257)	0.52	-546 (-1549, 456)	0.28	-312 (-658, 33)	0.076	-1282 (-3208, 643)	0.18
**7c,10c,13c,16c-Docosatetraenoic Acid (22:4n-6c)**	-3 (-6.5, 0.51)	0.09	-85 (-185, 14.8)	0.094	11 (-7.8, 29.8)	0.24	-81 (-177, 14)	0.095	-12 (-46, 22)	0.49	-86 (-275, 102)	0.36
**Trans**												

	**9t-Hexadecenoic Acid (16:1n-7t)**	0.6 (-7.3 8.5)	0.87	238 (19.5, 457)	0.033	6.8 (-35.3, 48.9)	0.74	126 (-98, 351)	0.26	78 (0.88, 155)	0.048	520 (116, 923)	0.012
	**6t-Octadecenoic Acid (18:1n-12t)**	-1.85 (-4.9, 1.23)	0.23	-98 (-183, -12)	0.026	-14.5 (30, 1.69)	0.078	-72 (-155, 11.9)	0.092	-11 (-41, 19)	0.46	-47 (-213, 118)	0.56
	**9t-Octadecenoic Acid (18:1n-9t)**	-1.8 (-6.4, 2.7)	0.43	-111 (-239, 16)	0.086	-0.06 (-24, 24)	0.99	-98 (-222, 25)	0.11	-8 (-52, 36)	0.71	-67 (-309, 176)	0.58
	**11t-Octadecenoic Acid (18:1n-7t)**	-2.5 (-6, 1.1)	0.17	45 (-57, 148)	0.37	15 (-3, 34)	0.1	17 (-82, 116)	0.73	10 (-24, 45)	0.55	48 (-144, 240)	0.62
	**9t,12t-Octadecadienoic Acid (18:2n-6t)**	2.9 (-29, 35)	0.85	1206 (329, 2083)	0.008	85 (-85, 256)	0.32	844 (-32, 1720)	0.059	260 (-49, 569)	0.098	1762 (94, 3430)	0.039
	**9c,12t-Octadecadienoic Acid (18:2n-6ct)**	0.23 (-7.12, 7.5)	0.95	58 (-150, 267)	0.58	17 (-22, 55)	0.39	-128 (-337, 80)	0.22	128 (61, 194)	0.0001	761 (413, 1109)	0.0001
	**9t,12c-Octadecadienoic Acid (18:2n-6tc)**	-4.75 (-12.1, 2.6)	0.2	-173 (-382, 36)	0.1	2.7 (-37, 42)	0.89	-176 (-378, 26)	0.087	-15 (-87, 57)	0.67	-42 (-441, 357)	0.83
**Other**													
	**9c,11t-Octadecadienoic Acid (18:2n-7c)**	-1.05(-10.7, 8.6)	0.83	289 (20.6, 556)	0.035	25.7 (-25.3, 77)	0.31	59.7 (-223, 343)	0.67	169 (79, 259)	0.0001	975 (501, 1448)	0.0001

Supplement Tables

Supplement Tables
